# Performance Enhancement for Indium-Free Metal Oxide Thin-Film Transistors with Double-Active-Layers by Magnetron Sputtering at Room Temperature

**DOI:** 10.3390/mi13112024

**Published:** 2022-11-19

**Authors:** Xingzhen Yan, Kaian Song, Bo Li, Yiqiang Zhang, Fan Yang, Yanjie Wang, Chao Wang, Yaodan Chi, Xiaotian Yang

**Affiliations:** Key Laboratory of Architectural Cold Climate Energy Management, Ministry of Education, School of Electronics and Computer, Jilin Jianzhu University, 5088 Xincheng Street, Changchun 130118, China

**Keywords:** thin film transistor, indium-free, metal oxide, double active layers

## Abstract

We prepared an indium-free metal oxide thin-film transistor (TFT) using a double-active-layers structure at room temperature. We changed the growth sequence of Al-doped zinc oxide (AZO) and zinc oxide (ZnO) double-active-layers on Si/SiO_2_ substrates by magnetron sputtering deposition to regulate the field-effect performance of TFTs. According to the analysis of field-effect properties before and after annealing in different atmospheres, the performance of TFT devices with ZnO/AZO/SiO_2_/Si double-active-layers was obviously better than that with single AZO or ZnO active layer and inverted AZO/ZnO/SiO_2_/Si double-active-layers in the device structure. The active layer with higher carrier concentration (AZO in this case) was closer to the dielectric layer, which was more favorable for carrier regulation in TFT devices. In addition, the annealed device had a lower on/off ratio (I_on_/I_off_), easier-to-reach on-state, and higher mobility. Furthermore, the performance of the devices annealed under vacuum condition was obviously better than that annealed under air atmosphere. The I_on_/I_off_ could reach 6.8 × 10^5^ and the threshold voltage was only 2.9 V.

## 1. Introduction

With the rapid development of display field, the demand for display drivers has gradually increased, and thin-film transistors (TFTs), as the key point of the electronic flat panel display industry, has attracted more attention [1,2,3]. The low power consumption and long-term stability requirements of current integrated circuits for TFTs is provoking researchers to consider the choice of materials and structure designs to achieve a tunable operational mechanism for a high-performance TFT device [4,5,6]. Recent studies have shown that, apart from changing the dielectric layer and electrodes, the choice of channel layers plays an important role in device performance optimization, mainly including organic semiconductors [7,8,9] and metal oxide materials [10,11,12,13,14]. Organic-based transistors have realized much larger scales of integration [7] and are close to being commercially available [15]. However, a major problem of organic devices is their instability, including electrical and thermal instability, and their degradation mechanisms affect the long-term operation of the devices [15,16]. The advantages of metal oxide TFT devices include transparency in the visible range, stability, and plurality of element combination with controllable doping, and they can be prepared by different scalable deposition technologies [17].

Metal oxide semiconductors can serve as channel layers and provide stable carrier regulation for TFTs that could be potentially used as the next commercial driver devices in an integrated circuit. Among oxide semiconductor channel layers, researchers have investigated the electrical and thermal stability to optimize the field-effect performance of TFT devices, such as pristine zinc oxide (ZnO) [18,19,20] and indium, tin, gallium, and other elements doped with ZnO [21,22,23,24,25,26,27,28]. Hosono groups gave us the deeper understanding of the amorphous oxides based on heavy metal oxides with the (*n* − 1)d^10^ns^0^ (*n* ≥ 4) electronic configuration for TFT materials [10]. For the design of TFTs’ structure, the double-active-layers have been adopted to improve the field-effect performance [29,30,31,32]. The double-active-layers structure was composed of one material (e.g., IGZO) or two materials (e.g., IZO and IGZO). One layer was the low-resistance IGZO or IZO with high carrier concentration and was close to the gate insulator. The defects in the channel and the interface between the active and insulating layer can be efficiently passivated by a low-resistance layer with high carrier concentration [32]. Among them, indium plays an important role in the high-performance semiconductor materials for metal oxide TFTs. However, the scarcity of indium materials will always restrict the subsequent large-scale preparation and wide application [33,34]. As an alternative candidate, indium or gallium-free active layers should attract more attention in future application processes. However, considering the effects of the indium element on mobility and carrier regulation and the gallium element on stability for active layers, an effective double-layer channel needs to be designed to achieve substitutions. Moreover, the morphology and grain boundary density of the semiconductor layer also affect the charge transport and field-effect performance of the thin-film transistor [35,36].

Doped metal oxides deposited by magnetron sputtering at room temperature are in the amorphous state. The oxides have strong iconicity, in which charges are transferred from metal to oxygen atoms. Additionally, the conduction band minimum of heavy metal cation oxides are mainly comprised of spherical ns-orbitals. Therefore, amorphous metal oxides can also have good electron transport properties [37]. Considering the current ZnO-doped elements, metal aluminum (Al) as a substitute doping can form carrier concentration regulation, which has the advantages of abundant element reserves and low temperature required for preparation. Furthermore, when ZnO materials are doped with Al ion donors (AZO), this active layer exhibits high carrier concentration, which provides a carrier source for field-effect regulation. At the same time, the high carrier concentration in the channel leads to a large off-state current, which will affect the on/off ratio (I_on_/I_off_) of TFT devices. Therefore, a ZnO layer was constructed on the AZO active layer to ensure a lower off-state current and avoid the influence of scattering center in carrier transport during device operation. Then, metal Al was evaporated on the ZnO layer as the source and drain electrodes to construct a bottom-gate and top-contact TFT structure. The double-active-layers TFT devices grown by rf magnetron sputtering had better electrical properties than the monolayer ZnO or AZO TFT structure. Moreover, the performance of TFT devices constructed by the double-active-layers after vacuum annealing (~200 °C) treatment was better than that of the device without treatment and exhibited a threshold voltage (V_T_) of 2.9 V, a saturation mobility (μ) of 0.01 cm^2^/Vs, and an I_on_/I_off_ value of 6.8 × 10^5^.

## 2. Materials and Methods

### 2.1. Preparation of TFT Devices

In this paper, Si/SiO_2_ (Si wafer with a thickness of 285 nm of SiO_2_ from HEFEI KEJING Materials Tech Co., Ltd., Hefei, China) slices were used as the bottom-gate electrode and dielectric layer. The Si/SiO2 sheets were cut into squares of size ~15 mm, which were, respectively, put into acetone, alcohol, and deionized water for ultrasonic cleaning for 10 min, and finally dried with nitrogen. AZO (2:98 wt %) and ZnO monolayer or double-layers structures were deposited on Si/SiO_2_ substrate by magnetron sputtering for 10 min. The growth conditions were set to a growth pressure of 8 mTorr, sputtering power of 100 W, and flow ratio of argon to oxygen 95:5.

**Figure 1:**
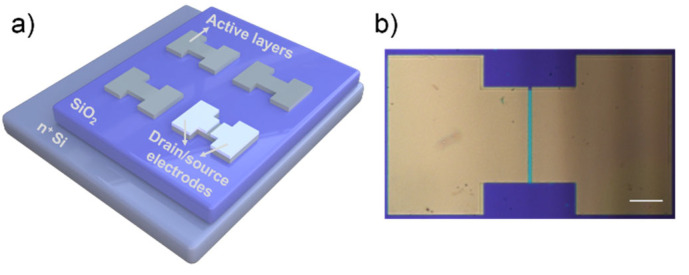
(**a**) Schematic diagram of fabrication of a metal oxide TFT device. (**b**) Microscope image of the channel pattern. The scale bar is 100 μm.

The substrate prepared with the active layer was placed in the gel homogenizer; the rotation speed and the duration were set at 3000 r/min and 30 s, respectively. Then, the photoresist was uniformly applied on the surface of the substrate. After that, the substrate was placed on a hot plate heated at 90 °C for 5 min, and the substrate was fixed on the patterned mask of the active layer for the first exposure. The UV radiation intensity and the exposure time were set to 420 mW/cm^2^ and 4 s, respectively. Additionally, the photoresist reacted with UV light was washed with 0.5% NaOH after the exposure, and the graphics were developed. The source and drain electrodes were formed by evaporation of aluminum metal via an electron beam evaporation system. The substrate heating temperature was set to 60 °C, and the thickness of electrodes was 50 nm. The channel length and width of all TFT devices were about 10 μm and 300 μm, respectively. A TFT device with metal oxide active layers and the structure consisting of bottom gate and top contact was constructed as shown in Figure 1. Then, the prepared samples were annealed to 200 °C for 5 min at a heating rate of 5 °C/s with different annealing atmospheres in a rapid annealing furnace. The annealing pressure of TFT devices under a vacuum environment was 0.5 hPa. The samples were removed from furnace at room temperature after annealing. The I_on_/I_off_ and V_T_ were estimated by extrapolating the linear portion of the (drain current (I_SD_))^1/2^ versus gate voltage (V_G_) curves at drain-source voltage (V_SD_) value of 20 V for all TFT devices.

### 2.2. Characterization

The active layers were prepared by rf magnetron sputtering (PVD75, Kurt. J. Lesker Company, Jefferson Hills, PA, USA). The electrodes and channel layers are graphically etched with a photolithography system (ABM/6/350/NUV/DCCD/M, ABM, Inc., San Francisco, CA, USA). The field effect characterizations were measured by a semiconductor parameter measuring instrument (B1500A, Keysight Technologies, Colorado Springs, CO, USA). The samples were annealed in different atmospheres using a vacuum rapid annealing furnace (RTP-100, UniTemp, Pfaffenhofen an der Ilm, Germany).

## 3. Results and Discussion

**Figure 2:**
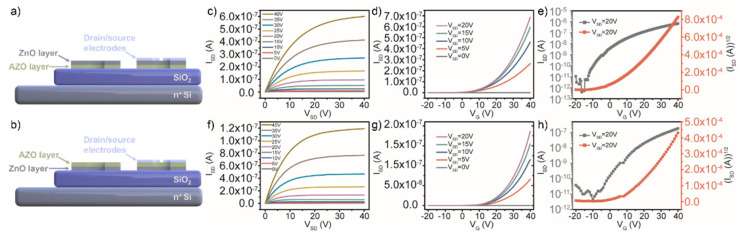
Schematic diagrams of cross-section structure of oxide TFTs with ZnO/AZO/SiO_2_/Si (**a**) and AZO/ZnO/SiO_2_/Si (**b**) double-active-layers. Output characteristics and transfer characteristics of TFTs with ZnO/AZO/SiO_2_/Si layers (**c**–**e**) and with AZO/ZnO/SiO_2_/Si layers (**f**–**h**).

The double-active-layers of ZnO and AZO layers were deposited on the Si/SiO_2_ substrate by magnetron sputtering; then, patterned channel layers and source leakage electrodes were obtained by lithography. The channel length and width of all TFT devices were about 10 μm and 300 μm, respectively. The deposition sequence of AZO with a higher doping concentration and ZnO with few impurity-scattering centers and more stability was modulated to verify the performance of double-active-layers TFT devices. The double-active-layers with the thickness of 32 ± 0.5 nm was formed by a 5 min deposition of each single-layer, either by preparing the AZO layer first and then the ZnO layer (ZnO/AZO/SiO_2_/Si) or by reversing the deposition sequence (AZO/ZnO/SiO_2_/Si), as shown in Figure 2a,b. Figure 2c,f show the drain current versus drain-source voltage (I_SD_-V_SD_) output characteristics of TFTs with ZnO/AZO/SiO_2_/Si and AZO/ZnO/SiO_2_/Si double-active-layers at V_G_ from 0 to 40 V, respectively. The curves show the typical n-type field-effect electrical properties with the clear transition from linear to saturation behavior with different deposition sequences of the channel layers. Figure 2d,g plot the typical transfer curves of these two TFT devices at different V_SD_ from 0 to 20 V. As shown in Figure 2e,h, the I_on_/I_off_ and the V_T_ estimated by extrapolating the linear portion of the (I_SD_)^1/2^ versus V_G_ curves at V_SD_ = 20 V of the two TFT devices with a double-active-layer structure can be calculated. The TFT with ZnO/AZO/SiO_2_/Si double-active-layers exhibited a V_T_ of 9.5 V, a field-effect mobility of 0.0040 cm^2^/Vs, and an I_on_/I_off_ of 6.3 × 10^4^. In contrast, the device with AZO/ZnO/SiO_2_/Si double-active-layers showed lower electrical performance, including a V_T_ of 14.3 V, a field-effect mobility of 0.0014 cm^2^/Vs, and an I_on_/I_off_ of 2.5 × 10^4^. The reason is that the AZO layer with more carrier concentration close to the insulation layer in devices can facilitate the regulation of more induced charges and obtain a lower operating voltage, thus reducing the power consumption of the TFT devices. In addition, the relatively stable ZnO layer can effectively ensure that the device has a lower off-state current. In addition, the subthreshold slopes (SS) of the TFT devices with ZnO/AZO/SiO_2_/Si and AZO/ZnO/SiO_2_/Si double-active-layers were 1.59 and 1.69 V/decade, respectively. The smaller SS value means that the ZnO/AZO/SiO_2_/Si TFTs switch from the off-state to the on-state more quickly and require a smaller voltage change.

**Figure 3:**
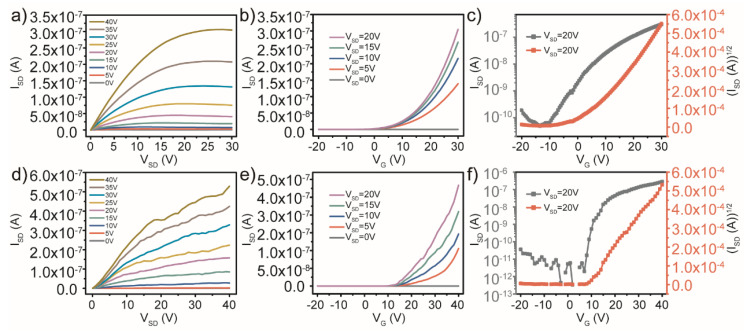
Output characteristics and transfer characteristics of TFTs with AZO single-active-layer (**a**–**c**) and with ZnO single-active-layer (**d**–**f**).

For better verification, the TFT devices with a single-active-layer of AZO and AZO deposited for 10 min (~32 ± 0.5 nm thickness) were compared with the double-active-layers device. The output characteristics and transfer characteristics of single-active-layer TFT devices are plotted in Figure 3. As shown in Figure 3a, the transition from the linear to the saturation regime and a good regulating effect on the I_SD_ are observable in the output characteristics of the TFTs with AZO single-active-layer under different V_G_. However, according to the performance of TFT devices derived from the transfer characteristics in Figure 3b,c, the value of I_on_/I_off_ of 4.7 × 10^3^ was relatively low. We know that the I_on_/I_off_ value determines the degree of antinoise signal interference of TFT devices. The reason is that the single-layer AZO with higher carrier concentration as the active layer makes the TFT device retain a higher off-state current. The field-effect mobility was also reduced to 0.0029 cm^2^/Vs by the introduction of more carrier scattering centers. However, the TFT device with a single-layer ZnO without other metal cations as an active layer does not exhibit the hard saturation and current regulation effects in the output characteristic and transfer characteristic curves, as shown in Figure 3d–f. Considering integral electrical performance of devices, the double-active-layers ZnO/AZO/SiO_2_/Si TFTs were elected for following optimization and discussion due to the higher I_on_/I_off_ and field-effect mobility.

**Figure 4:**
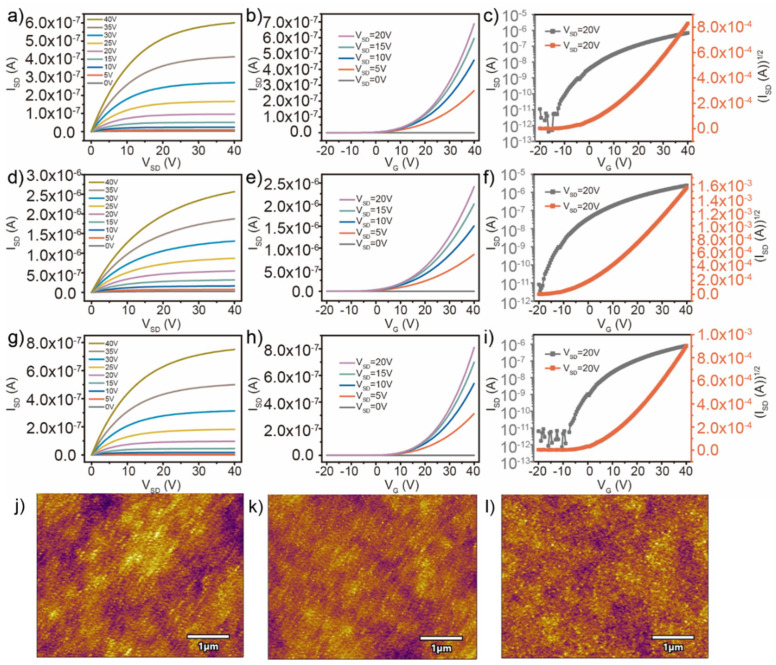
Output characteristics and transfer characteristics of TFTs with ZnO/AZO/SiO_2_/Si double-active-layers before (**a**–**c**) and after annealing in vacuum environment (**d**–**f**) and in air (**g**–**i**). Atomic force microscope images of the ZnO/AZO channel layers without (**j**) and with annealing treatment in vacuum environment (**k**) and in air (**l**).

The deposition conditions of both single- and double-active-layers discussed above were all completed at room temperature. Annealing treatment was required to release stress effects caused by atomic stacking in the channel layers. In order to avoid the gathering of functional groups on the surface of channel layers during the annealing process, the electrical properties of the double-active-layers ZnO/AZO/SiO_2_/Si TFTs before and after 5 min annealing at 200 °C in a vacuum environment and air atmosphere are compared in Figure 4. As shown in Figure 4d, the TFTs after vacuum annealing exhibit a good current regulation ability. In addition, it was found that the I_SD_ value was larger than that of nonannealed samples under the same V_G_, which was due to the improved carrier mobility caused by the decrease in defects and the increase in regulated carrier concentration in conductive channels. The field-effect performance, including a V_T_ of 2.9 V, a field-effect mobility of 0.010 cm^2^/Vs, and an I_on_/I_off_ of 6.8 × 10^5^ derived from the transfer characteristics, was significantly improved compared with the TFT devices before vacuum annealing treatment. In contrast, the field-effect performance, including a V_T_ of 9.1 V, a field-effect mobility of 0.0043 cm^2^/Vs, and an I_on_/I_off_ of 1.4 × 10^5^ of the device after annealing in air atmosphere, was not significantly improved compared with that before annealing, as shown in Figure 4i. Although the stress defects in active layers were improved, the adsorption of functional groups on the channel surface will lead to the creation of acceptor-like surface states [38]. So, vacuum annealing can avoid introducing excessive surface defects to affect the TFT device performance. Furthermore, an annealing treatment can effectively increase the surface particle size and reduce the surface roughness so as to improve the scattering problem during carrier injection and migration. The variation of the double-active-layer ZnO/AZO/SiO_2_/Si surface roughness before and after annealing in a vacuum environment and air was analyzed by atomic force microscope in Figure 4j–l. The surface roughness of the ZnO/AZO channel layers without annealing treatment was 1.183 nm, and the roughness of the double-active layers was decreased to 0.913 nm and 1.057 nm after annealing in a vacuum environment and air, respectively. The reduction of interface roughness can further improve the electrical transport properties of TFTs. Finally, the field-effect parameters of the TFT devices with various active layers and annealing atmosphere are summarized in Table 1

## 4. Conclusions

We investigated a double-active-layers TFT device with a bottom-gate and top-contact (drain/source electrodes) structure and discussed the effect of different double-active-layers stacking modes on TFT properties and the advantages over a comparable device with a single-active-layer. The ZnO/AZO/SiO_2_/Si double layers had the advantage of higher carrier concentration and lower off-state current, which improved the regulation ability of I_SD_ in the conductive channel of TFT devices. In addition, we introduced vacuum annealing treatment to improve the stress defects caused by atomic packing and to enhance the field-effect performance of the device. The optimized double-active-layers TFT exhibited a V_T_ of 2.9 V, a field-effect mobility of 0.010 cm^2^/Vs, and an I_on_/I_off_ of 6.8 × 10^5^. However, compared with the full-fledged devices, this ZnO/AZO/SiO_2_/Si TFT still has a certain gap in the field-effect performance. In the next step, we will improve the electrical properties by improving and modifying the interface contact quality between the double-active-layers.

## Figures and Tables

**Table 1:** The field-effect parameters of the TFTs with various active layers and annealing atmosphere.

Type	Threshold Voltage (V)	On/Off Ratio
AZO/ZnO/SiO_2_/Si	14.3	2.5 × 10^4^
ZnO/AZO/SiO2/Si	9.5	6.3 × 10^4^
After annealing in vacuum	2.9	6.8 × 10^5^
After annealing in air	9.1	1.4 × 10^5^
